# 

**Published:** 1921-12

**Authors:** 


### The Library of the Bristol Medico-Chirurgical Society.

*The following donations have been received since the publication of the List in September.*

*December 31st,* 1921.

| | | |
|---|---|---|
| Dr. Carey Coombs (1) | .. .. .. .. | 2 volumes. |
| Dr. Shingleton-Smith (2) | .. .. .. .. | 1 volume. |
| Dr. J. O. Symes (3) | .. .. .. .. .. | 1 ,, |
| Mr. C. H. Walker (4) | .. .. .. .. | 3 volumes. |

Unbound periodicals have been received from Dr. Carey Coombs, Dr. Newman Neild, and Dr. W. A. Smith.

### THE ONE HUNDRED AND FIFTH LIST OF BOOKS.

The figures in brackets refer to the figures after the names of the donors, and show by whom the volumes were presented. The books to which no such figures are attached have either been bought from the Library Fund or received through the *Journal*.

| | | |
|---|---|---|
| **Bourne, A. W.** .. | *Synopsis of Midwifery* .. .. .. 2nd Ed. | 1921 |
| **Groves, E. W. H.** .. | *Modern Methods of Treating Fractures.* 2nd Ed. | 1921 |
| **Morris, Sir M.** [Ed.] | *Dictionary of Practical Medicine,* 3 vols. .. | 1921 |
| **Murrell, W.** .. .. | *What to do in cases of Poisoning* 12th Ed. | 1921 |
| **Osborn, H.** .. .. | *Golden Rules of Dental Mechanics* .. .. .. .. | [N.D.] |
| **Platt, H.** .. .. | *Peripheral Nerve Injuries* .. .. .. .. .. .. | 1921 |
| **Rogers, Sir L.** .. | *Bowel Diseases in the Tropics* .. .. .. | [1921] |
| **Sherren, J.** .. .. | *Surgery of the Stomach and Duodenum* .. .. | 1921 |
| **Sorapure, V. E.** [Ed.] | *The Oxford Index of Therapeutics* .. .. .. | [1921] |
| **Thompson, D. W.** | *On Growth and Form* .. .. .. .. .. (2) | 1917 |
| **Wilde, P.** .. .. | *Physiology of Gout, Rheumatism and Arthritis* | 1921 |
| **Wilson, R. M.** .. | *Symptoms and Treatment of Circulatory Disease* .. .. .. .. .. .. .. .. .. | [1921] |

### TRANSACTIONS, REPORTS, JOURNALS, &c.

| | | |
|---|---|---|
| American Journal of Surgery .. | Vols. XXXIII., XXXIV. | 1919–20 |
| Boston Medical and Surgical Journal .. .. | Vols. CLXXV., CLXXVI., CLXXVIII, CLXXXI.—CLXXXIII. | 1916–20 |
| British Journal of Dermatology .. .. | Vols. XXX.—XXXII. | 1918–20 |
| British Journal of Ophthalmology .. | (4) Vols. I., II., IV. | 1917–20 |

Gazette hebdomadaire des Sciences med. de Bordeaux Tom. XXXV. XXXVI. 1914–15
Johns Hopkins Hospital Bulletin .. .. .. .. .. Vol. XXXI. 1920
Journal of Experimental Medicine .. Vols. XXXII., XXXIII. 1920–21
Journal of Laryngology .. .. .. .. .. .. .. Vol. XXXV. 1920
Journal of Medical Research .. .. .. .. .. .. Vol. XLI. 1919–20
Journal of Tropical Medicine.. .. .. Vols. XIX.—XXIII. 1916–20
Medical Clinics of North America.. .. .. .. (3) Vol. III. 1919–20
Medical Science .. .. .. .. .. .. .. Vols. II.—IV. 1920–21
Quarterly Journal of Medicine .. .. (1) Vols. XIII., XIV. 1919–21
Royal Society of Medicine, Proceedings of .. .. .. Vol. XIII. 1920

---

### Obituary.

### Local Medical Notes.

---

University of Bristol.—Markham Skerritt Prize has been awarded to Sir J. Herbert Parsons, C.B.E., F.R.C.S.

Students of the University have recently passed the following examinations :—

M.D. Lond.—*Medicine :* E. W. Wade.

M.S. Lond.—W. Salisbury, M.D.

Dip. Trop. Med., Cantab.—W. W. Pratt, D.P.H. Lond.

F.R.C.S.—*Primary Examination :* Gladys Llewelyn, B.Sc., J. A. James, J. R. Nicholson-Lailey.

M.B., Ch.B. Bristol.—*2nd Examination, Part I. :* Helen M. Aldwinckle, E. C. Bernard, Winifred P. Brinckman, G. L. Feneley, F. W. T. Hughes, T. P. Lalonde, C. B. Perry, L. B. Phillips, E. Southam, A. B. Vincent. *Anatomy only :* O. J. Dolland. *Organic Chemistry only :* J. F. O. Bodman, B. J. Boulton, Muriel E. Drew, W. L. Sleight.

Conjoint Board.—*Anatomy and Physiology :* W. O. H. Evans, J. Hughes. *Surgery :* J. A. Prichard. *Midwifery :* Hilda E. Reynolds, J. A. Prichard, M. Critchley.

L.M.S.S.A.—J. L. Delicati, C. F. Long.

L.D.S., R.C.S. Eng.—1*st Examination, Part III. :* (*a*) H. H. Watkins ; (*b*) W. G. Smith, F. H. Woolley, A. J. C. Constance.

### INDEX.

Abdominal conditions, Some problems in surgical treatment of—F. Fraser, 1.
Acids in infection, The rôle of dilute—Drs. I. Walker Hall and A. D. Fraser, 158.
American College of Surgeons Library, 167.

Ballance, Dr. H. S., Obituary Notice of, 127.

Blood chemistry in disease, 37.

**Books, Reviews of**—
Air sickness—Cruchet and Moulinier, 55.
Aitchison, Dr. R. S.—A medical handbook, 56.
Anæsthetics—D. W. Buxton, 113.
Anatomy—H. Gray, 52.
Anatomy, Guide to—E. D. Ewart, 98.
Anxiety hysteria—Dr. C. H. Rixon, 112.
Austen, E. E.—The house fly, 114.
Ballance, Sir C. A.—Surgery of the heart, 57.
Bardswell, Dr. N.—Handbook for tuberculosis workers, 101.
Barton, G. A. H.—Backwaters of lethe, 109.
Blackham, R. J.—Military sanitation, 117.
Bone formation, On—Dr. M. Jansen, 105.
Bristow, Dr. W. R.—Treatment of joint and muscle injuries, 105.
Broca, A.—Ligations and amputations, 108.
Buckmaster, Dr. G. A.—Course of practical physiology, 98.
Bulletins et memoires de la Société Anat., 110.
Buxton, D. W.—Anæsthetics, 113.
Clinical methods—Drs. R. Hutchison and H. Rainey, 99.
Collis, Dr. E. L.—The industrial clinic, 110.
Cope, Dr. Z. — Surgical aspects of dysentery, 108.
Copestake, B. M. G.—Massage, 164.
Cruchet and Moulinier—Air sickness, 55.
Daukes, Dr. S. H.—Barrier charts for health officers, 115.
Diagnosis, Differential—Dr. H. French, 102.
Diathermy—Dr. C. Saberton, 166.
Doctor's manual, The—Dr. A. H. Hart, 56.
Dysentery, Surgical aspects of—Dr. Z. Cope, 108.
Electricity, Medical—Dr. H. L. Jones, 116.
Ewart, E. D.—Guide to anatomy, 98.
Flint, Dr. H. L.—The heart: old and new views, 101.
Fly, The house—E. E. Austen, 114.
Fortescue-Brickdale, Dr. J. M.—Pharmacology and treatment for nurses, 51.

**Books, Reviews of** (*continued*)—
French, Dr. H.—Differential diagnosis, 102.
Fuller, Sir B.—The science of ourselves, 163.
Gonococcal infections—Dr. N. Lumb, 106.
Gray, H.—Anatomy, 52.
Greenwood, Dr. W. O.—Scopolamine-morphine, 165.
Groves, E. W. H.—Synopsis of surgery, 108.
Gulland, M. A.—Nursing, 114.
Hart, Dr. A. H.—The doctor's manual, 56.
Hay, Dr. J.—Graphic methods in heart disease, 102.
Health officers, Barrier charts for—Dr. S. H. Daukes, 115.
Heart, The: old and new views—Dr. H. L. Flint, 101.
Heart disease, Graphic methods in—Dr. J. Hay, 102.
Heart, Surgery of the—Sir C. A. Ballance, 57.
History of Surgery in Great Britain—Dr. G. Parker, 94.
Horder, Sir T.—Medical notes, 101.
Human machinery, The care of the—Dr. R. M. Wilson, 106.
Hurst, Dr. A. F.—The psychology of the special senses,' 163
Hutchison and H. Rainey, Drs. R—Clinical methods, 99.
Ibbotson, W.—Atlas of the sensory cutaneous nerves, 115.
Industrial clinic, The—Dr. E. L. Collis, 110.
Infectious diseases—C. B. Ker, 54.
James, Dr. S. P.—Malaria, 51.
Jansen, Dr. M.—On bone formation, 105.
Joint and muscle injuries—Dr. W. R. Bristow, 105.
Jones, Dr. H. L.—Medical electricity, 116.
Journal of neurology and psychotherapy, 109.
Keith, Dr. A.—Menders of the maimed, 103.
Kenwood, Dr. H. R.—Public health laboratory work, 113.
Ker, C. B.—Infectious diseases, 54.
Lang, Dr. W. D.—British Mosquitoes, 165.
Laurens, Dr. G.—Oto-rhino-laryngology, 112.
Lethe, Backwaters of—G. A. H. Barton, 109.
Ligations and amputations—A. Broca, 108.
Lumb, Dr. N.—Gonococcal infection in the male, 106.
Malaria—Dr. S. P. James, 51.
Manson's tropical diseases, 99.
Martindale's extra pharmacopœia, 111.
Massage—B. M. G. Copestake, 164.
Masters, Dr. W. E.—Essentials of tropical medicine, 54.
Medical annual, The, 108.
Medical handbook, A—Dr. R. S. Aitchison, 56.
Medical notes—Sir T. Horder, 101.
Medicine, Manual of—Dr. A. S. Woodwark, 98.

**Books, Reviews of** (*continued*)—
Medicine, Synopsis of—Dr. H. L. Tidy, 98.
Menders of the maimed—Dr. A. Keith, 103.
Mental illnesses, Functional—Dr. R. G. Rows, 49.
Military Sanitation—R. J. Blackham, 117.
Miller, Dr. H. C.—Functional nerve disease, 54.
Mosquitoes, British—Dr. W. D. Lang. 165.
Nerve disease, Functional—Dr. H. C. Miller, 54.
Nervous diseases, Diagnosis of—Sir J. P. Stewart, 53.
Nursing—M. A. Gulland, 114.
Obstetrics, Practical—Drs. E. H. Tweedy and G. T. Wrench, 111.
Operative Surgery—A. Thomson and A. Miles, 57.
Oto-rhino-laryngology—Dr. G. Laurens, 112.
Parker, Dr. G.—The early history of surgery in Great Britain, 94.
Paton, Dr. D. N.—Essentials of human physiology, 97.
Peripheral nerve injuries—H. S. Souttar, 103.
Pharmacology and treatment for nurses—Dr. J. M. Fortescue-Brickdale, 51.
Pharmacopœia, Martindale's extra, 111.
Pharmacopœia of the Queen's Hospital for children—116.
Physiology, Essentials of human—Dr. D. W. Paton, 97.
Physiology, Course of practical—Dr. G. A. Buckmaster, 98.
Physiology, The new—Dr. A. R. Short, 97.
Psychology of the special senses, The—Dr. A. F. Hurst, 163.
Public health laboratory work—Dr. H. R. Kenwood, 113.
Rats, Prevention and destruction of, 115.
Rixon, Dr C. H. L.—Anxiety hysteria, 112.
Rows, Dr. R. G.—Functional mental illnesses, 49.
Saberton, Dr. C.—Diathermy, 166.
Science of ourselves, The—Sir B. Fuller, 163.
Scopolamine-morphine — Dr. W. O. Greenwood, 165.
Sensory cutaneous nerves, Atlas of—W. Ibbotson, 115.
Short, Dr. A. R.—The new physiology, 97.
Souttar, H. S.—Injuries of the peripheral nerves, 103.
Stewart, Sir J. P.—Diagnosis of nervous diseases, 53.
Surgery, Synopsis of—E. W. H. Groves, 108.
Surgery in Great Britain, The early history of—Dr. G. Parker, 94.
Syphilis, Atlas of—Drs. C. F. White and W. H. Brown, 55.
Thomson and A. Miles, A.—Operative surgery, 57.
Tidy, Dr. H. L.—A synopsis of medicine, 98.
Treatment, Dictionary of—Sir W. Whitla, 53.
Tropical medicine, Essentials of—Dr. W. E. Masters, 54.
Tropical diseases, Manson's, 99.
Tuberculosis workers, Handbook for—Dr. N. Bardswell, 101.
Tweedy and G. T. Wrench, Drs. E. H.—Practical Obstetrics, 111.

**Books, Reviews of** (*continued*)—
White and W. H. Brown, Dr. C. F.—Atlas of syphilis, 55.
Whitla, Sir W.—Dictionary of treatment, 53.
Wilson, Dr. R. M.—The care of the human machinery, 106.
Woodwark, Dr. A. S.—Manual of medicine, 98.

Bone injuries, Repair of—E. W. H. Groves, 142.
Breath, Shortness of, Discussion on, 67.
Bristol Medico-Chirurgical Society, 66.

Cervix uteri, Incurable carcinoma of, 47.
Clarke, Dr. C.—Report on medicine, 37.
Clarke, Dr. R. C.—The teething myth, 28.
Coombs, Dr. C. P., Death of, 167.

Duodenal ulcer, Treatment of perforating, 46.

Editorial notes, 58, 118, 167.
Empyema, Progress in the treatment of—Dr. J. A. Nixon, 82.
Eye diseases and quackery—C. H. Walker, 129.

Fortescue-Brickdale, Dr. J. M., Obituary notice of, 63, 69.
Fox (Long) Lecture—E. W. H. Groves, 142.
Fraser, Drs. I. W. Hall and A. D.—The rôle of dilute acids in infection, 158.
Fraser, F.—Some problems in surgical treatment of abdominal conditions, 1.

Gastric function, 40.
Gastric ulcer, Treatment of perforating, 46.
Gordon, Dr. R. G.—The present position of psychotherapy, 11, 66.

Groves, E. W. H.—The Long Fox lecture on the repair of bone injuries, 142.
Gynæcology, Report on—Dr. R. S. Statham, 47.

Hall and A. D. Fraser, Drs. I. W.—The rôle of dilute acids in infection, 158.
Harty, Dr. J. P. I.—Remarks on sinus phlebitis and thrombosis, 73.
Homœopathic hospital buildings, 125.
Hospitals, Financial problems of, 58.

Infection, The rôle of dilute acids in—Drs. I. W. Hall and A. D. Fraser, 158.
Infirmary, Royal, Almoner's report, 122.

Library, Bristol Medical, Growth of, 168.
Library of the Bristol Medico-Chirurgical Society, 64, 125, 170.
Local medical notes, 72, 128, 172.
Loveday, Prof. T., 169.

Medicine, report on.—Dr. C. Clarke, 37.

Nixon, Dr. J. A.—Progress in the treatment of Empyema, 82.

Obituary Notices, 69, 127, 171.
Orr-Ewing, Dr. H. J.—Case of multiple serositis of unusual distribution, 90.
Owen, Sir I., Resignation of, 62; Dinner to, 169.

Paraplegia, Retention of urine in, 43.
Pick's disease, Case of—Dr. H. J. Orr-Ewing, 90.
Postgraduate teaching, 61, 121.
Prostate, Carcinoma of, 44.
Psychotherapy, The present position of—Dr. R. G. Gordon, 11, 66.

Quackery in relation to diseases of the eye—C. H. Walker, 129.

Rossiter, Dr. G. F., Obituary notice of, 171.

Sanatorium for tuberculosis in Bristol, 124.
Serositis of unusual distribution, Case of—Dr. H. J. Orr-Ewing, 90.
Siepmann memorial prize, 123.
Sinus phlebitis and thrombosis—Dr. J. P. I. Harty, 73.
Statham, Dr. R. S.—Report on gynæcology, 47.
Surgery, Report on—Dr. J. Swain, 43.
Swain, Dr. J.—Report on Surgery, 43.

Teething myth, The—Dr. R. C. Clarke, 28.

University appeal fund, 118.
Urine, Retention of, in paraplegia, 43.

Walker, C. H.—Some phases of quackery in relation to diseases of the eye, 129.

J. W. Arrowsmith Ltd.
Publishers
Bristol

### *PUBLICATIONS RECEIVED.*

From John Bale, Sons and Danielsson Ltd., London :

*Health in few Words.* By H. R. Firth, D.P.H. 1921. Price 2d.

From Castle and Company Ltd., London :

*The Dictionary of Practical Medicine.* By Many Writers. Edited by Sir Malcolm Morris, K.C.V.O., Frederick Langmead, M.D., and Gordon M. Holmes, C.M.G. C.B.E., M.D. Three Volumes. 1921. Price £5 5s. net.

From Dr. Gibbon Fitzgibbon, Dublin :

*Clinical Report of the Rotunda Hospital.* 1917–20. [Reprint.]

*A Method of Inducing Labour.* By Gibbon Fitzgibbon, M.D. 1921. [Reprint.]

*Prevention and Treatment of Ophthalmia Neonatorum.* By Gibbon Fitzgibbon, M.D. 1920. [Reprint.]

From Henry Frowde and Hodder & Stoughton, London :

*Manual of Physiology.* By H. Willoughby Lyle, M.D. and David De Souza, M.D. Second Edition. 1921. Price 21s. net.

*Bowel Diseases in the Tropics.* By Sir Leonard Rogers, C.I.E., M.D. [1921.] Price 30s. net.

*The Oxford Index of Therapeutics.* Edited by Victor E. Sorapure, M.B., Ch.B., F.R.C.S.E. [1921.] Price £2 2s. net.

*The Clinical Study of the Early Symptoms and Treatment of Circulatory Disease in General Practice.* By R. M. Wilson, M.B., Ch.B. [1921.] Price 15s. net.

From W. Green and Son, Edinburgh :

*An Introduction to Dermatology.* By Norman Walker, LL.D., M.D. Seventh Edition. 1922. Price 21s. net.

From H. K. Lewis and Co. Ltd., London ;

*Lectures on the Surgery of the Stomach and Duodenum.* By James Sherren, C.B.E., F.R.C.S. 1921. Price 4s. 6d. net.

*What to do in cases of Poisoning.* By Wm. Murrell, M.D. Twelfth Edition, revised by P. Hamill, M.D. 1921. Price 4s. 6d. net.

From John Wright & Sons Ltd., Bristol :

*Synopsis of Midwifery.* By Aleck W. Bourne, B.A., M.B., B.Ch. Second Edition. 1921. Price 15s. net.

*The Surgery of the Peripheral Nerve Injuries of Warfare.* By Harry Platt, M.S., F.R.C.S. 1921. Price 4s. net.

*The Physiology of Gout, Rheumatism, and Arthritis.* By Percy Wilde, M.D. 1921. Price 12s. 6d. net.

*On Modern Methods of Treating Fractures.* By Ernest W. Hey Groves, M.S., M.D., F.R.C.S. Second Edition. 1921. Price 30s. net.

*Golden Rules of Dental Mechanics.* By Harold Osborn. [n.d.] Price 5s. net.

### Surgical Operations. Instruments & Appliances.

BY

WILLIAM IBBOTSON, M.R.C.S., L.R.C.P.

**IN TWO PARTS:**

PART I.

### Surgical Operations:

Classified Lists, with full particulars of Instruments and Appliances required for each operation and explanatory notes.

PART II.

### Illustrations of over 450 Instruments & Appliances alphabetically arranged.

Size $7\frac{1}{2} \times 5$. Bound in limp cloth.

Price **5/-** net. By post **5/3**

---

*Of all Booksellers or of*

**THE SCIENTIFIC PRESS LTD.,**

**28/29 SOUTHAMPTON STREET, STRAND, LONDON, W.C.2.**

